# Availability of two essential medicines for mental health in Bangladesh, the Democratic Republic of Congo, Haiti, Nepal, Malawi, Senegal, and Tanzania: Evidence from nationally representative samples of 7958 health facilities

**DOI:** 10.7189/jogh.12.04063

**Published:** 2022-08-01

**Authors:** Muhammad A Rahman, Yusuf Babaye, Amritha Bhat, Pamela Y Collins, Christopher G Kemp

**Affiliations:** 1 Information School, University of Washington, Seattle, Washington, USA; 2 International Training and Education Center for Health-Malawi, Lilongwe, Malawi; 3 Department of Global Health, University of Washington, Seattle, Washington, USA; 4 Department of Psychiatry and Behavioral Sciences, University of Washington, Seattle, Washington, USA; 5 Department of International Health, Johns Hopkins Bloomberg School of Public Health, Baltimore, Maryland, USA

## Abstract

**Background:**

Access to effective mental health services in low- and- middle income countries (LMICs) is limited, leading to a substantial global treatment gap. Amitriptyline, an anti-depressant, and diazepam, an anxiolytic drug, are classified as essential medications by the World Health Organization (WHO). They are the only psychotropic medications whose availability in health facilities is documented as part of Service Provision Assessment surveys. Our objective was to characterize the availability of these medicines in seven countries.

**Methods:**

We pooled nationally representative data from Service Provision Assessment surveys of health facilities conducted in Bangladesh, Democratic Republic of Congo (DRC), Haiti, Malawi, Nepal, Senegal, and Tanzania, from 2012 to 2018. We estimated the distribution and determinants of facility-level amitriptyline and diazepam availability in each country.

**Results:**

We analysed data from 7958 health facilities. An estimated 8.2% of facilities had amitriptyline and 46.1% had diazepam on the day of assessment. There was significant heterogeneity in both amitriptyline and diazepam availability across countries and within countries across facility characteristics. Multivariable models indicated that hospitals, faith-based and private-for-profit facilities, facilities with more staff, and facilities with more technological resources were more likely to have each medicine, relative to primary care facilities, public sector facilities, facilities with fewer staff, and facilities with fewer technological resources, respectively.

**Conclusion:**

Our results indicate limited availability of amitriptyline in health facilities in these seven LMICs. Diazepam is much more commonly available than amitriptyline. Efforts to narrow the global treatment gap for mental health – and especially to integrate mental health services into primary care in LMICs – will be limited without the availability of essential medicines like amitriptyline. Efforts to expand purchasing, distribution, and capacity-building in the appropriate use of essential mental health medicines in LMICs are warranted.

Depression is among the top drivers of disability of the past 30 years, affecting over 264 million people worldwide [1,2]. Yet, particularly in low- and- middle income countries (LMICs), treatment and support services are often underfunded or absent, meaning there is a global gap in the availability of and access to depression treatment [3]. Only an estimated 1 in 23 people in LMICs receive minimally adequate treatment for depression [4,5]. On average less than 1% of national health budgets of LMICs are spent on mental health [6]. To narrow the global treatment gap for depression and other common mental health conditions, it will be critical to allocate additional resources to strengthen capacity for decentralized, community-based mental health services and to increase efforts to integrate mental health care into primary care [7].

Evidence-based mental health treatment includes psychotropic medications as well as psychotherapy. Antidepressant medications are the first line of treatment for severe depression and are indicated alongside psychotherapy for moderate to severe depression [8]. Amitriptyline is a tricyclic antidepressant commonly prescribed for major depressive disorder and other depressive disorders [9]. It also can be used to treat anxiety disorders, pain, and less commonly insomnia and attention-deficit/hyperactivity disorder [10]. Diazepam is a type of benzodiazepine which can be used to treat generalized anxiety disorder, insomnia, seizures, social phobia, and panic disorder, though it is no longer prioritized as the first line of treatment in some countries and has potentially serious side effects [11]. The World Health Organization (WHO) has declared both amitriptyline and diazepam to be essential medications for depressive and anxiety disorders, respectively [12,13]. Diazepam is also the WHO essential medicine for sleep disorders [14]. Antidepressants such as amitriptyline are frequently prescribed by primary care providers in the treatment of depression their availability is important for the effective integration of mental health and primary care services [15,16]. Facility-level availability of these medicines is therefore one useful benchmark for the ability of a health system to provide essential services for depression, anxiety, and other mental disorders, especially at the primary care level.

There is limited evidence concerning the distribution of availability of essential psychotropic medicines in health facilities across LMICs. The Demographic and Health Surveys (DHS) Program conducts Service Provision Assessments (SPAs) in selected countries to assess the capacity of health facilities and understand the availability of various preventive and treatment services. The SPA was reformed in 2012 to begin collecting information regarding treatment for non-communicable diseases, including the availability of amitriptyline and diazepam. Our objective was to analyse available SPA data to understand the distribution and determinants of facility-level amitriptyline and diazepam availability in seven LMICs. Our analysis was limited to amitriptyline and diazepam, as the SPA did not ask about the availability of other essential psychotropic medicines.

## METHODS

### Data sources

Facility-level data came from standard SPAs conducted from 2012 through 2018: Bangladesh (2017) [17], Democratic Republic of Congo (DRC, 2017-18) [18], Haiti (2017-18) [19], Malawi (2013-14) [20], Nepal (2015) [21], Senegal (2012-16) [22], and Tanzania (2014-15) [23]. Haiti had two SPAs (2013 and 2017-18) in this period, as did Bangladesh (2014 and 2017), and Senegal has had one every year since 2012 through 2016. The most recent survey was used for Haiti and Bangladesh, and all the most recently surveyed unique facilities in Senegal through 2016 were included. The Nepal, DRC, and Tanzania SPAs used nationally representative facility samples. The Haiti and Malawi SPAs were censuses of all health facilities in each country. The Bangladesh SPA was a sample representative of all public facilities and all private hospitals, but it excluded small private facilities. The Senegal SPAs were national samples of formal-sector health facilities. All sampled facilities reporting that they stored medications were included in this analysis. True GPS coordinates of each facility were used when available.

Community-level variables came from household DHS conducted immediately before or after each of the included SPA surveys. GPS coordinates of each DHS sampling cluster centroid were included, though these were randomly displaced up to 5 km, with 1% displaced up to 10 km.

**Table 1** presents a brief description of each country and statistics related to population mental health, including the availability of human resources for mental health. Country-level characteristics were taken from the WHO ATLAS 2017 [24]. Malawi and DRC had no available 2017 data and therefore the 2014 version was used [25]. Global Burden of Disease (2017) estimates of the prevalence of depressive disorders in each country were also used in addition to World Bank estimates of per capita GDP [26.

**Table 1:** Socioeconomic and mental health-related indicators for included countries

	Bangladesh	Democratic Republic of Congo	Haiti	Malawi	Nepal	Senegal	Tanzania
**Population (2020) **	170 492 922	92 378 000	11 743 017	19 431 566	30 378 055	17 223 497	59 441 988
**Region **	South Asia	Central Africa	Caribbean	East Africa	South Asia	West Africa	East Africa
**GDP per capita ($USD 2019)**	$302571.25	$47319.62	$8498.98	$7666.7	$30641.38	$23578.08	$63177.07
**World Bank classification **	Lower-middle income	Low-income	Low-income	Low-income	Low-income	Lower-middle income	Lower-middle income
**Psychiatrists (rate per 100 000 population) [** **17** **]**	0.13	0.08	0.07	0.01	0.36	0.2	0.06
**Mental health nurses (rate per 100 000 population) [** **17** **]**	0.87	0.5	0.2	0.22	0.56	0.27	0.36
**Psychologists (rate per 100 000 population) [** **17** **]**	0.12	0.02	0.56	0.02	0.52	0.02	0.01
**Prevalence of major depressive disorder, % of total population **	2.79%	2.25%	1.85%	1.72%	2.15%	1.70%	1.93%
**Mental health policy? (WHO ATLAS) [** **17** **]**	Yes	Yes	Yes	Yes	Yes	Yes	Yes
**Stand alone law for mental health?**	Yes	Not reported (NR)	No	Yes	No	Yes	Yes
**Government total expenditure on mental health as % of total government health expenditure**	0.50%	NR	1.62%	NR	NR	NR	4.00%
**Amitriptyline and diazepam on the essential drug list?**	Yes	Yes	Yes	Yes	Yes	Yes	Yes
**Service provision assessment sampling approach**	Nationally representative, excluding small private facilities	Nationally representative	Census	Census	Nationally representative	Nationally representative of formal-sector health facilities	Nationally representative

### Key variables

Our primary outcomes of interest were binary, indicating whether each health facility had valid, un-expired amitriptyline or diazepam available on the day of observation. We explored facility- and community-level covariates that we hypothesized would explain the availability of each medicine in the sampled facilities. Covariates at the facility level included: numbers of full-time staff, facility ownership, facility type, the availability of key structural resources such as central electricity, improved water, and a functioning computer. We also considered the centrality of each health facility with respect to national health system leadership, estimated using the land-based travel time from each facility to the respective nation’s Health Ministry headquarters. These headquarters are in the national capital of each country. Notably, Tanzania has two de facto capitals and two Health Ministry headquarters (Dodoma and Dar es Salaam); we estimated travel time from each facility to the headquarters in its nearest capital. We derived travel time estimates using the 2015 Malaria Atlas Project global friction surface [27]. At the community level, we adjusted for the average wealth of households nearest to each health facility. We derived this variable by taking the mean of wealth indices for households sampled in each matched DHS at the level of the sampling cluster and linking that mean to each health facility based on the shortest Euclidean distance between each facility and a sampling cluster.

### Analysis

We calculated sample-weighted proportions of medicine availability to explore variation by country, type, and ownership. We then used generalized linear mixed effects models to explore the determinants of amitriptyline and diazepam availability using multiple levels of variable adjustment. All models used the binomial family and logit link and included random country-specific intercepts to adjust for clustering. Sampling weights were not used in the multivariate analysis, as unweighted estimates are preferable when the purpose is to examine structural relationships within a sample [28]. Complete case analysis was used. Results are displayed as adjusted odds ratios (aOR) with 95% confidence intervals (95% CI). Data analysis was done in R software version 4.0.5 (Foundation for Statistical Computing, Vienna, Austria, 2020) [29].

## RESULTS

**Figure 1:**
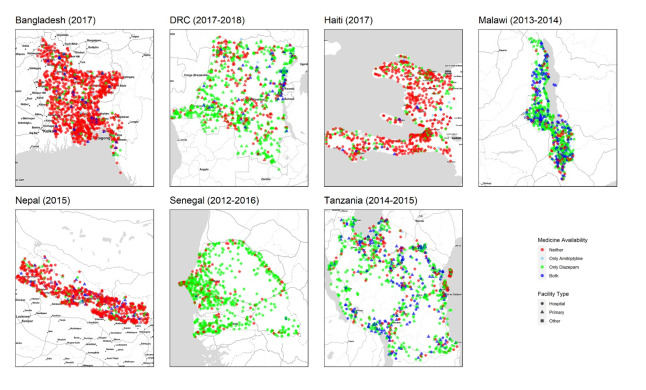
Geographic distribution of availability of Amitriptyline and Diazepam, by facility type, from nationally representative facility samples in seven countries (n = 7958).

Data from 7958 facilities met our inclusion criteria and were available for analysis. **Figure 1** presents the geographical distribution of sampled health facilities. **Table 2** presents descriptive statistics, stratified by country. 8.2% of all included facilities had amitriptyline on the day of observation, while 46.1% of facilities had diazepam. There was substantial heterogeneity in availability by country. For example, 0.2% of facilities in Senegal had amitriptyline, while 36.2% of facilities in Malawi had amitriptyline. Similarly, 5.0% of facilities in Bangladesh had diazepam, while 78.1% of facilities in Malawi had diazepam.

**Table 2:** Sample-weighted facility descriptive statistics from nationally representative facility samples in seven countries (n = 7958)

	Overall	Bangladesh	DRC	Haiti	Malawi	Nepal	Senegal	Tanzania
n = true sample size	7958	1496	1368	999	964	918	1043	1170
n = weight sample size	8048.5	1513	1359.9	999	963.8	941.1	1107.4	1164.3
Facility characteristics:								
**Number of staff (%):**								
0-5	49.2	90.2	27.3	30.8	32.1	61.0	25.6	64.6
6-10	22.8	3.9	42.4	27.3	15.0	26.0	32.0	15.5
11-25	17.7	1.6	22.0	23.9	34.2	7.2	27.3	13.7
25+	10.3	4.3	8.3	17.9	18.7	5.9	15.2	6.2
Facility ownership (%):								
**Government/public**	71.4	93.5	61.5	34.3	48.5	92.5	86.7	73.3
**NGO/private not-for-profit**	7.2	0.7	2.5	17.0	12.8	2.7	6.1	12.7
**Private-for-profit**	12.8	3.4	17.8	29.8	21.8)	4.8	3.8	12.4
**Mission/faith-based**	8.6	2.3	18.2	18.8	16.9	0.1	3.5	1.5
**Facility type (%):**								
**Hospital**	7.0	2.7	10.0	13.1	11.6	8.0	2.4	4.0
**Primary**	92.6	96.9	90.0	86.9	87.9	90.2	97.6	96.0
**Other**	0.4	0.5	0.0	0.0	0.5	1.9	0.0	0.0
**Facility resources:**								
**Central supply of electricity (%)**	13.6	17.1	2.3	5.1	23.0	9.6	23.4	15.8
**Improved water source (%)**	83.3	93.0	58.0	81.8	97.0	89.8	94.2	74.4
**Improved sanitation facilities (%)**	61.2	79.1	30.5	61.2	36.8	81.2	93.7	43.0
**Functioning computer (%)**	34.1	67.2	10.5	46.4	33.8	22.9	31.8	19.4
**Internet available (%)**	33.4	57.7	4.1	59.8	40.5	11.6	44.8	14.3
**Available room with visual and auditory privacy (%)**	89.4	78.7	87.5	91.0	95.9	78.2	94.3	93.8
Facility location:								
**Travel time to central ministry of health, hours (%):**								
0-5	64.4	95.9	13.0	92.5	91.0	69.5	53.7	32.8
6-10	16.8	4.1	9.1	7.5	8.8	24.0	23.1	43.0
11+	18.8	0.0	77.9	0.0	0.2	6.4	23.3	24.2
Psychiatric medicines:								
**Amitriptyline available (%)**	8.2	2.4	3.0	3.8	36.2	7.5	0.2	10.7
**Diazepam available (%)**	46.1	5.0	63.7	23.3	78.1	8.9	76.1	73.7

Most sampled facilities (71.4%) were government-owned/public. Almost half (49.2%) were staffed by 0-5 full-time staff members. Notably, facilities in Bangladesh (90.2%), Nepal (61.0%), and Tanzania (64.6%) predominantly had 0-5 full-time staff, while the staff numbers were more evenly distributed in other countries. Most were primary care facilities (92.6%) rather than hospitals (7.0%).

Most facilities had an improved water source (83.3%) and available rooms with visual and auditory privacy (89.4%). However, relatively few facilities were connected to a central supply of electricity (13.6%) or had a functioning computer (34.1%) or available internet (33.4%). In Haiti and DRC, 5.1% and 2.3% of the sampled facilities were connected to a central supply of electricity, respectively. Bangladesh (67.2%) was the only country with most facilities having functioning computers. Similarly, only Haiti (59.8%) and Bangladesh (57.7%) had functioning internet at most facilities. Improved sanitation facilities were available in most health care facilities in all countries except Tanzania (43.0%), Malawi (36.8%), and DRC (30.5%).

Most facilities (64.4%) were estimated to be within five hours travel of the central Health Ministry of each country. DRC facilities were substantially more geographically dispersed than facilities in other countries, with 77.9% estimated to be over 11 hours travel from the central Ministry in Kinshasa.

**Figure 2:**
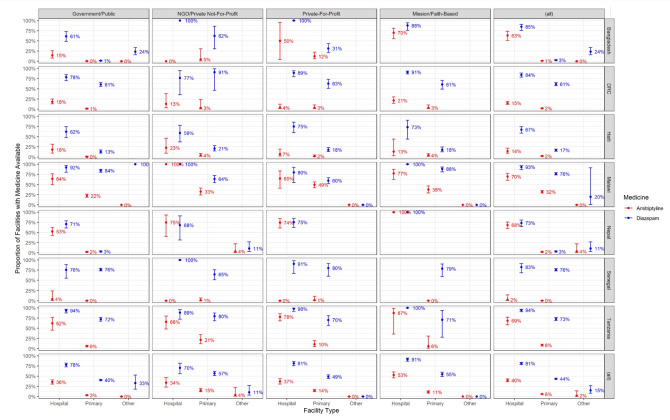
Sample-weighted proportions of facilities with available Amitriptyline and Diazepam, stratified by facility type, ownership, and country, from nationally representative facility samples in seven countries (n = 7958).

**Figure 2** provides weighted estimates of availability of each medication by country, availability of each medication by country, ownership, and type of facility. Only 6% of primary care facilities report having amitriptyline, compared to 40% of hospitals and 2% of other facilities. 44% of primary care facilities reported having diazepam, compared to 81% of hospitals and 15% of other facilities. Public primary care facilities were the least likely to have amitriptyline (3%) and diazepam (40%) while faith-based hospitals were the most likely to have amitriptyline (53%) and diazepam (91%).

Similar facility level characteristics were associated with the likelihood of facilities having amitriptyline and with facilities having diazepam (**Table 3**). Facilities with more staff were significantly more likely to have both amitriptyline and diazepam compared to facilities with fewer staff, whereas primary care facilities were substantially less likely than hospitals to have amitriptyline (aOR = 0.29, 95% CI = 0.22-0.37) or diazepam (aOR = 0.35, 95% CI = 0.27-0.45). Compared to public facilities, private-for-profit facilities (aOR = 2.55, 95% CI = 2.00-3.25) and faith-based facilities (aOR = 2.18, CI = 1.67-2.84) were more likely to have amitriptyline. They were also more likely to have diazepam, though the relative difference was less. Several facility resources were consistently associated with both amitriptyline and diazepam availability, including having a functioning computer, having internet, and having an available room with visual and auditory privacy.

**Table 3:** Associations between facility characteristics and availability of amitriptyline and diazepam from nationally representative facility samples in seven countries (n = 7958)

		Amitriptyline availability			Diazepam availability												
	**Overall**	**Not available**	**Available**	**aOR**	**95% CI**			***P*-value**	**Not available**		**Available**		**aOR**		**95% CI**		***P*-value**	
**Facility characteristics**																		
Number of staff (%)																		
0-5	49.2	51.7	21.6	–	–			–	65.1		30.6		–		–		–	
6-10	22.8	23.5	14.1	1.6	1.16-2.20			**0.004**	20.8		25.1		1.77		1.46-2.14		**<0.001**	
11-25	17.7	16.8	27.3	2.31	1.75-3.04			**<0.001**	9.8		26.8		3.49		2.86-4.25		**<0.001**	
25+	10.3	8.0	37	3.45	2.48-4.78			**<0.001**	4.2		17.5		5.11		3.93-6.65		**<0.001**	
Facility ownership (%):																		
Government/public	71.4	74.4	37.9	–	–			–	77.1		64.7		–		–		–	
NGO/private not-for-profit	7.2	6.6	14.5	1.58	1.17-2.13			**0.003**	5.8		8.8		1.18		0.92-1.53		0.199	
Private-for-profit	12.8	11.5	28.2	2.55	2.00-3.25			**<0.001**	11		15		1.37		1.11-1.70		**0.004**	
Mission/faith-based	8.6	7.6	19.4	2.18	1.67-2.84			**<0.001**	6.1		11.5		1.44		1.12-1.83		**0.004**	
Facility type (%)																	
Hospital	7	4.6	34.1	–	–	–	2.4			12.4		–		–		–	
Primary	92.6	95	65.8	0.29	0.22-0.37	**<0.001**	97			87.5		0.35		0.27-0.45		**<0.001**	
Other	0.4	0.4	0.1	0.02	0.00-0.17	**<0.001**	0.6			0.1		0.09		0.03-0.25		**<0.001**	
Facility resources:																	
Central supply of electricity (%)	13.6	12.6	24.4	1.04	0.85-1.28	0.693	12			15.5		0.9		0.75-1.09		0.297	
Improved water source (%)	83.3	82.3	94.5	1.08	0.77-1.52	0.641	84			82.4		1		0.81-1.22		0.963	
Improved sanitation facilities (%)	61.2	61.1	62.2	1.26	1.02-1.55	**0.034**	65.4			56.1		0.91		0.77-1.07		0.256	
Functioning computer (%)	34.1	31.5	62.8	2.22	1.76-2.80	**<0.001**	36.5			31.2		1.4		1.15-1.69		**0.001**	
Internet available (%)	33.4	31.3	56.9	1.42	1.15-1.75	**0.001**	34.9			31.7		1.34		1.11-1.61		**0.002**	
Available room with visual and auditory privacy (%)	89.4	88.8	94.8	1.61	1.11-2.34	**0.012**	84.5			94.1		1.44		1.13-1.82		**0.003**	
Facility location:																	
Travel time to central ministry of health, hours (%):																	
0-5	64.4	63.2	77	–	–	–	3087.3 (74.3)			1851.5 (52.6)		–		–		–	
6-10	16.8	17.1	13.8	0.99	0.77-1.27	0.935	562.4 (13.5)			725.9 (20.6)		1.07		0.87-1.31		0.522	
11+	18.8	19.7	9.3	1.32	0.94-1.84	0.106	504.0 (12.1)			940.3 (26.7)		1.49		1.15-1.92		**0.002**	
Mean neighboring household wealth, quartiles (%):																	
First	24.7	25.3	18.4	–	–	–	1052.2 (25.3)			842.5 (23.9)		–		–		–	
Second	26.2	26.70	20.9	0.92	0.72-1.19	0.545	1101.3 (26.5)			911.0 (25.9)		1.08		0.89-1.31		0.437	
Third	24.7	24.6	26.1	1.09	0.85-1.40	0.474	1036.9 (25.0)			859.9 (24.4)		0.78		0.64-0.95		**0.013**	
Fourth	24.3	23.4	34.7	0.96	0.73-1.24	0.736	962.8 (23.2)			904.3 (25.7)		0.73		0.59-0.91		**0.005**	
Intercept				0.02	0.00-0.08	**<0.001**						0.73		0.24-2.25		0.588	
**Random effects**																	
σ^2^				3.29								3.29					
τ_00_				2.86 _country_								1.98 _country_					
ICC				0.46								0.38					
N				7 _country_								7 _country_					
Observations				6015								6015					
Marginal R^2^ / conditional R^2^				0.246 /0.596								0.205/0.504					

aOR – adjusted odds ratio, CI – confidence interval, ICC – intra-cluster correlation

Travel time to each facility from the Health Ministry headquarters was not consistently associated with medicine availability, though facilities more than 11 hours away were more likely to have diazepam available (aOR = 1.49, 95% CI = 1.15-1.92). Similarly, the average wealth of nearby communities was not consistently associated with medicine availability, though facilities near communities in the two highest wealth quartiles were significantly less likely to have diazepam.

## DISCUSSION

We assessed the availability of two essential medications for the treatment of depression and anxiety across large, nationally representative samples of health facilities in seven LMICs. Amitriptyline and diazepam are on the essential list of medications for every country analysed [30-36]. We found that most health facilities in these settings do not have amitriptyline available, though many do have diazepam. Hospitals are more likely to have both medications; amitriptyline is significantly lacking in primary care facilities.

Our results align with prior research suggesting that LMICs tend to allocate public mental health-related funding to large, centrally located psychiatric hospitals and mental health inpatient units in general hospitals. A study on service readiness of health facilities in Bangladesh, Haiti, Kenya, Malawi, Namibia, Nepal, Rwanda, Senegal, Uganda, and Tanzania found that deficiencies in medications and diagnostic capacity were more variable between hospitals and health centres/clinics within the same country, compared to across countries [37]. We found that hospitals, regardless of country, were more likely to carry amitriptyline compared to primary care clinics and other facilities. We also noted that this difference was especially pronounced in public sector facilities and was less pronounced in not-for-profit or faith-based facilities; this may be explained by the fact that these facilities at least partially fund their own services [5,38].

Two challenges to narrowing the global treatment gap include integrating core aspects of mental health services into routine primary health care and improving the supply of effective psychotropic drugs for mental disorders [39]. Psychotherapy services should ideally be complemented by appropriate and available pharmacotherapy [40], and primary-care-based treatment of depression and panic disorder using older drugs like amitriptyline has been found to be more cost-effective than the use of newer drugs in LMICs [41]. Amitriptyline and comparable drugs therefore continue to have an important potential role in LMICs with fewer resources, though the cost of generic selective serotonin reuptake inhibitors (SSRIs) has fallen since 2004 [42]. Ultimately, improving the management of common mental health conditions will require a significant increase in psychotropic treatment availability, especially in primary care settings [43].

We also observed substantial heterogeneity across countries, with over one third of facilities in Malawi and one in ten in Tanzania having amitriptyline available compared to nearly none in Senegal. One possible explanation, given the generalized HIV epidemic in Malawi and Tanzania, is that there may have been particular investment in their primary care depression services by their governments or global donors given the established link between untreated depression and poor HIV-related outcomes [44-46].

It should be noted that the side effect burden of older antidepressants is higher than newer SSRIs leading to high rates of early discontinuation [47]. Therefore, it is imperative that we expand assessment of the distribution and availability of SSRIs and other psychiatric medications in LMICs. This would also allow more comprehensive analysis of the availability of essential mental health services.

The availability of these two medications serves as one indicator for the readiness of health systems to provide essential services for mental health. A 2014 report of the Institute of Medicine, Improving Access to Essential Medicines for Mental, Neurological, and Substance Use Disorders in Sub-Saharan Africa, explored barriers and opportunities to essential medicine access, focusing on demand, selection, supply chains, financing and pricing [48]. Patterns of help-seeking for mental disorders, supply dynamics, and adequacy of care each influence demand for essential medications. Notably, the designation of medication as ‘essential’ is based on safety and effectiveness data, with the recognition that “subsequently licensed compounds may be safer or more effective [49].” Ensuring the proper use and distribution of these medicines when they are available is as important as ensuring their supply. A recent study found that diazepam was widely available in Tanzania, yet the majority of facilities improperly dispensed it without a prescription [50]. The relatively greater supply of diazepam in almost half of the sampled facilities raises the question of how providers are using this medicine while amitriptyline – considered a core treatment in these settings – is not readily available. A Brazilian study noted that diazepam without an antidepressant is frequently prescribed for anxiety and insomnia accompanying depressive symptoms [51]. It is likely facilities also carry diazepam for other medical conditions, such as muscle spasms, seizure disorders or alcohol withdrawal, although determining exactly why facilities stock diazepam and when they dispense it is difficult to ascertain.

Pharmaceutical care and supply chain are also essential to ensuring access to quality medical products and constant availability of health commodities. These include good dispensing practices and pharmacovigilance as well effective procurement, storage, and distribution. The supply chains for medications to treat depression and anxiety and other mental disorders are challenged by low levels of use in some settings, as well as lack of provider or patient awareness of the treatment option [52]. Although travel time did not affect medication availability in our data, it may affect patient access to treatment.

Our findings are reinforced by our use of large, nationally representative samples of health facilities pooled across seven countries. Nonetheless, several limitations should be acknowledged. First, the SPA surveys provide a snapshot of services available at each facility on a single assessment day. We were unable to assess the regularity of medicine availability, the actual accessibility of medicines by patients receiving care at these facilities, or the capacity of clinic staff to appropriately prescribe these medicines. Second, our analysis was limited to amitriptyline and diazepam, as the SPA did not ask about the availability of other essential psychiatric medicines. Furthermore, we only included valid (ie, non-expired) medicines in our analysis. Third, our findings are limited to the seven countries in the sample. And fourth, both diazepam and amitriptyline are also used to treat non-mental health conditions, and the SPA does not document how a facility is using its supply of diazepam or amitriptyline.

Nonetheless, this study has substantial policy implications, especially for efforts to strengthen the capacity of primary care facilities to deliver effective mental health care. Our findings suggest that the few LMICs primary care facilities can provide medicines like amitriptyline to patients on demand; this appears to be especially true of public primary care facilities. Therefore, increased investment by government health systems and by donors in the purchasing and distribution of psychiatric medications to public sector primary care facilities is warranted. Our results also suggest a need to better understand the current usage of available mental health medicines in these facilities, including diazepam, and to intervene in that usage where it is inappropriate.

## CONCLUSION

We have identified a severe shortage in amitriptyline at health facilities in seven LMICs. Diazepam is much more readily available, possibly due to its use for several conditions other than depression and anxiety. These medicines are concentrated in private facilities and hospitals and are much less likely to be available in public primary health care facilities. Increased investment in purchasing and distribution of essential medicines for common mental health conditions is warranted as part of a comprehensive effort to deliver quality evidence-based mental health services through the health system.
